# PRIORITIZING GERIATRIC EDUCATION FOR HEALTH CARE PROVIDERS

**DOI:** 10.1093/geroni/igae098.3113

**Published:** 2024-12-31

**Authors:** Lana Brown, Wanda Morrison, Julie Smith, Dennis Sullivan

**Affiliations:** Central Arkansas Veterans Healthcare System, North Little Rock, Arkansas, United States; Central Arkansas Veterans Healthcare System, North Little Rock, Arkansas, United States; Central Arkansas Veterans Healthcare System, North Little Rock, Arkansas, United States; Central Arkansas Veterans Healthcare System, North Little Rock, Arkansas, United States

## Abstract

Within the United States, there is a deficit of Geriatricians providing care for older adults, and this deficit will only grow as the population continues to age, meaning all health care providers will be responsible for the care of older adults. A learner needs assessment is a systematic approach to examining what individuals or groups need to learn in relation to health care practice. A learner needs assessment is a core element of designing effective, targeted geriatric educational interventions. In 2022, a brief six question online survey was distributed to all health care providers within the South Central Veterans Affairs (VA) Health Care Network to determine the top geriatric education needs. Within the survey, provider demographics requested were primary discipline and primary work setting (inpatient/outpatient). Respondents were requested to choose at least 5 out of 50 topics provided that would be the most useful for future geriatric education. Responses were received from 143 health care providers with the majority of the responses being from social workers 59(41.3%) and nurses 45(31.5%). There were 103 health care providers from outpatient areas and 40 from inpatient areas. The top geriatric education topics requested were mental health (50.4%), Alzheimer’s disease/dementia (48.3%), legal issues (38.5%), chronic pain (35.0%), depression (32.2%), and management of chronic conditions (30.1%). Experts were recruited to provide geriatric education on the highest priority requested topics. Providing geriatric education improves care for older adults ensuring health care providers are prepared to care and advocate for this diverse and often vulnerable population.

